# Genetic Deletion of the Clathrin Adaptor GGA3 Reduces Anxiety and Alters GABAergic Transmission

**DOI:** 10.1371/journal.pone.0155799

**Published:** 2016-05-18

**Authors:** Kendall R. Walker, Amit Modgil, David Albrecht, Selene Lomoio, Philip G. Haydon, Stephen J. Moss, Giuseppina Tesco

**Affiliations:** 1 Alzheimer's Disease Research Laboratory, Department of Neuroscience, Tufts University School of Medicine, 136 Harrison Avenue, St 328A, Boston, Massachusetts, 02111, United States of America; 2 AstraZeneca-Tufts Laboratory for Basic and Translational Neuroscience, Department of Neuroscience, Tufts University School of Medicine, Boston, Massachusetts, 02111, United States of America; 3 AstraZeneca Neuroscience Innovative Medicines, Cambridge, Massachusetts, 02421, United States of America; 4 Department of Neuroscience, Physiology and Pharmacology, University College, London, WC1E 6BT, United Kingdom; 5 Department of Neuroscience, Tufts University School of Medicine, 136 Harrison Avenue, Boston, Massachusetts, 02111, United States of America; Nathan Kline Institute and New York University Langone Medical Center, UNITED STATES

## Abstract

Golgi-localized γ-ear-containing ARF binding protein 3 (GGA3) is a monomeric clathrin adaptor that has been shown to regulate the trafficking of the Beta-site APP-cleaving enzyme (BACE1), which is required for production of the Alzheimer’s disease (AD)-associated amyloid βpeptide. Our previous studies have shown that BACE1 is degraded via the lysosomal pathway and that depletion of GGA3 results in increased BACE1 levels and activity owing to impaired lysosomal trafficking and degradation. We further demonstrated the role of GGA3 in the regulation of BACE1 *in vivo* by showing that BACE1 levels are increased in the brain of GGA3 null mice. We report here that GGA3 deletion results in novelty-induced hyperactivity and decreased anxiety-like behaviors. Given the pivotal role of GABAergic transmission in the regulation of anxiety-like behaviors, we performed electrophysiological recordings in hippocampal slices and found increased phasic and decreased tonic inhibition in the dentate gyrus granule cells (DGGC). Moreover, we found that the number of inhibitory synapses is increased in the dentate gyrus of GGA3 null mice in further support of the electrophysiological data. Thus, the increased GABAergic transmission is a leading candidate mechanism underlying the reduced anxiety-like behaviors observed in GGA3 null mice. All together these findings suggest that GGA3 plays a key role in GABAergic transmission. Since BACE1 levels are elevated in the brain of GGA3 null mice, it is possible that at least some of these phenotypes are a consequence of increased processing of BACE1 substrates.

## Introduction

Golgi-localized γ-ear-containing ARF binding proteins (GGAs) are monomeric clathrin adaptors that are recruited to the trans-Golgi network (TGN) by the Arf1-GTPase. Three GGAs (GGA1, 2 and 3) have been identified in mammals. They consist of four distinct segments: a VHS (VPS27, Hrs, and STAM) domain that binds the acidic di-leucine sorting signal, DXXLL; a GAT (GGA and Tom1) domain which binds Arf:GTP and ubiquitin; a hinge region which recruits clathrin; and a GAE (gamma-adaptin ear homology) domain which exhibits sequence similarity to the ear region of γ-adaptin and recruits a number of accessory proteins. GGAs are necessary for the sorting of acid hydrolases to the lysosomes. Newly synthesized acid hydrolases modified with mannose 6-phosphate groups bind to mannose 6-phosphate receptors (MPRs). In addition to MPRs, other cargo molecules bind to the VHS domain of GGAs via the DXXLL motif [1]. However, several sources of evidence support a unique role for GGA3 in the trafficking of ubiquitinated cargoes to lysosomes [2–5].

GGAs have been shown to bind the Beta-site APP-cleaving enzyme (BACE1) [6–9], a membrane-tethered member of the aspartyl proteases that has been identified as β-secretase [10–12]. The serial proteolysis of the amyloid precursor protein (APP) by β- and γ-secretase [13] results in the generation of a ~4kDa peptide termed Aβ, the main component of senile plaques accumulating in the brain of subjects affected by Alzheimer’s disease. Our previous studies have shown that BACE1 is degraded via the lysosomal pathway [14] and that depletion of GGA3 results in increased BACE1 levels and activity owing to impaired lysosomal trafficking and degradation [15, 16]. Moreover, we found that, unexpectedly, direct binding of GGA3 VHS domain to BACE1 via the di-leucine motif is not necessary for this regulation. Instead, we demonstrated that GGA3 interaction with ubiquitin is essential for the regulation of BACE1 levels [16]. We further demonstrated the role of GGA3 in the regulation of BACE1 *in vivo* by showing that BACE1 levels are increased in the brain of GGA3 null mice [17]. We also determined that depletion of GGA3 naturally occurs following caspase activation both in cellular models of apoptosis and in rodent models of stroke and traumatic brain injury [15, 17]. More importantly, we discovered that levels of GGA3 are decreased and inversely correlated with BACE1 levels in post-mortem AD brains [15].

GGA3 is highly expressed in the brain and in neurons [17, 18], however the function of GGA3 in the brain remains to be clarified. Thus, we performed a behavioral analysis of GGA3 null mice and found that GGA3 deletion results in novelty-induced hyperactivity and decreased anxiety-like behaviors. Given the pivotal role of GABAergic transmission in the regulation of anxiety-like behaviors [19], we performed electrophysiological recordings in hippocampal slices and found increased phasic and decreased tonic inhibition in the dentate gyrus granule cells (DGGC). Moreover, we found that the number of inhibitory synapses is increased in the dentate gyrus of GGA3 null mice in further support of the electrophysiological data. Thus, the increased GABAergic transmission is a leading candidate mechanism underlying the reduced anxiety-like behaviors observed in GGA3 null mice. All together these findings suggest that GGA3 plays a key role in GABAergic transmission. Since we have previously shown that BACE1 levels are elevated in the brain of GGA3 null mice, it is possible that at least some of these phenotypes are a consequence of increased processing of BACE1 substrates.

## Materials and Methods

### Animals

The generation of Gga3-/- (GGA3 KO) mouse line has been already described [17]. Briefly, the strain was created by microinjection of E14Tg2a.4 from 129P2/OlaHsd embryonic stem (ES) cells generated by BayGenomics (see http://baygenomics.ucsf.edu). The gene-trap vectors used within BayGenomics contain a splice-acceptor sequence upstream of a reporter gene, β-geo (a fusion of β-galactosidase and neomycin phosphotransferase II). These vectors insert randomly into introns. Chimeric males were mated to C57BL/6J females (Jackson laboratories) and the resulting heterozygous male was purchased. The mice used in these experiments have been backcrossed for 10 generations in C57BL/6J genetic background. Mice were housed under standard conditions and food and water were available ad libitum. All animal experiments were carried out with the approval of Tufts University Institutional Animal Care and Use Committees.

### Behavioral Analyses

All behavioral testing was conducted in the Tufts University CNR Behavior Core Facility. A total number of 21 GGA3KO mice (14 females, 7 males) and 28 GGA3WT mice (17 females, 11 males) were subjected to a battery of behavioral tests at 8–10 weeks of age (denoted as behavioral paradigm 1). In paradigm one, as per institutional guidelines to reduce unnecessary use of animals, we tested animals on a battery of tests starting with the least invasive/stressful and moving to more stressful tests. Of note, an inter-test interval of at least 24 hours was always employed. Behavioral testing paradigm 1 (in order of tests performed): Open Field Testing, Elevated-Plus-Maze, Light/Dark Exploration, Home cage monitoring, Marble Burying, Rotarod, Contextual and Cued Fear Associated Memory. Based upon results obtained from this battery of tests we subjected an additional n = 14 GGA3KO mice (7 females, 7 males) and 14 GGA3WT mice (7 females, 7 males) to behavioral tests to evaluate additional aspects of working memory and spatial reference memory in these lines (denoted as behavioral paradigm 2 to clarify that these mice were not subjected to the behavioral tests in paradigm 1). Behavioral testing paradigm 2 (in order of tests performed): Morris Water Maze. Based upon the reduced anxiety witnessed in elevated plus maze and light/dark test we were interested in the observing if there were any differences in learned helplessness between the genotypes so we ran the Porsolt Forced Swim Test, however this test could not be included with the tests in paradigm 2 which already included a swim task that allowed escape. Thus, an additional cohort of n = 15 GGA3KO mice (7 females, 8 males) and 23 GGA3WT mice (11 females, 12 males) were subjected to the Porsolt Forced Swim Test (this is denoted as behavioral testing paradigm 3 to indicate that these mice were not tested in either of the two other batteries of behavioral tests). All mice were acclimated to a reverse 12:12hrs light:dark cycle (behavioral testing performed during dark hours) for 2 weeks prior to the initiation of behavioral testing in order to test during the most active phase for the mouse. Statistical analysis of the behavioral data by two-way ANOVA indicated no genotype x gender interaction, but a genotype effect (Table 1) as a result, pooled sex data is presented in this manuscript.

**Table 1:** 

	Two way ANOVA							*t*-test	
**Circadian Activity Monitoring (24hr)**	**Interaction**		**Genotype**		**Gender**		**GGA3KO n = 21**	**GGA3WT n = 28**	**p-value**
Distance moved (cm)	F (1, 45) = 0.05615	P = 0.8138	F (1, 45) = 0.03905	P = 0.8442	F (1, 45) = 0.7117	P = 0.4033	79044 ±3754	80814 ± 3667	n.s.
Total rest time (min)	F (1, 45) = 0.2238	P = 0.6384	F (1, 45) = 0.4954	P = 0.4851	F (1, 45) = 6.030	P = 0.0180	858.45 ± 6.75	862.2 ± 5.6	n.s.
**Rotarod** (4-40rpm acceleration)	**Interaction**		**Genotype**		**Gender**		**GGA3KO n = 21**	**GGA3WT n = 28**	**p-value**
Max. rpm	F (1, 45) = 0.4495	P = 0.5060	F (1, 45) = 2.346	P = 0.1326	F (1, 45) = 2.637	P = 0.1114	30.4 ± 1.2	32.3 ± 0.9	n.s.
**Open Field Testing**	**Interaction**		**Genotype**		**Gender**		**GGA3KO n = 21**	**GGA3WT n = 28**	**p-value**
Total Path length (cm)	F (1, 45) = 0.4416	P = 0.5097	F (1, 45) = 21.06	P < 0.0001	F (1, 45) = 0.2502	P = 0.6194	2900 ± 108	2313 ± 73	p<0.0001
Time in Center (sec)	F (1, 45) = 3.244	P = 0.0784	F (1, 45) = 2.855	P = 0.0980	F (1, 45) = 0.6826	P = 0.4131	46.3 ± 4	34 ± 3	p = 0.0262
Time in Periphery (sec)	F (1, 45) = 3.244	P = 0.0784	F (1, 45) = 2.855	P = 0.0980	F (1, 45) = 0.6826	P = 0.4131	248 ± 6	267 ± 3	p = 0.0262
% Path length in center (cm)	F (1, 45) = 1.636	P = 0.2074	F (1, 45) = 2.408	P = 0.1277	F (1, 45) = 0.0001789	P = 0.9894	0.219 ± 0.013	0.184 ± 0.011	p = 0.0518
**Elevated Plus Maze**	**Interaction**		**Genotype**		**Gender**		**GGA3KO n = 21**	**GGA3WT n = 28**	**p-value**
Total Path length (cm)	F (1, 45) = 0.4808	P = 0.4916	F (1, 45) = 0.8244	P = 0.3687	F (1, 45) = 0.2141	P = 0.6458	1752.9 ± 36.4	1827.1 ± 49.6	n.s.
Open Arm entries	F (1, 45) = 0.6029	P = 0.4415	F (1, 45) = 10.06	P = 0.0027	F (1, 45) = 1.740	P = 0.1938	10.3 ± 0.5	7.5 ± 0.7	p = 0.0017
Time spent in Open Arms (%)	F (1, 44) = 0.06187	P = 0.8047	F (1, 44) = 13.39	P = 0.0007	F (1, 44) = 1.351	P = 0.2513	0.28 ± 0.02	0.16 ± 0.02	p = 0.0003
Distanced moved in Open Arm (cm)	F (1, 45) = 0.3624	P = 0.5502	F (1, 45) = 11.63	P = 0.0014	F (1, 45) = 2.202	P = 0.1448	518.3 ± 34.1	333.4 ± 38.7	p = 0.0012
Closed Arm entries	F (1, 45) = 1.084	P = 0.3035	F (1, 45) = 4.623	P = 0.0370	F (1, 45) = 0.06996	P = 0.7926	12.7 ± 0.7	14.5 ± 0.6	p = 0.0285
Time spent in Closed Arms (%)	F (1, 45) = 1.005	P = 0.3215	F (1, 45) = 7.841	P = 0.0075	F (1, 45) = 6.943	P = 0.0115	0.53 ± 0.02	0.63 ± 0.02	p = 0.0024
**Light Dark Transition**	**Interaction**		**Genotype**		**Gender**		**GGA3KO n = 21**	**GGA3WT n = 28**	**p-value**
Total Path length (cm)	F (1, 45) = 0.8595	P = 0.3588	F (1, 45) = 2.250	P = 0.1406	F (1, 45) = 0.01106	P = 0.9167	1986 ± 95	1755 ± 80	p = 0.0689
Light chamber entries	F (1, 45) = 0.006880	P = 0.9343	F (1, 45) = 8.843	P = 0.0047	F (1, 45) = 0.4417	P = 0.5097	16.1 ± 0.9	11.8 ± 1	p = 0.0029
Time spent in light chamber (%)	F (1, 45) = 0.1490	P = 0.7013	F (1, 45) = 3.317	P = 0.0752	F (1, 45) = 0.9826	P = 0.3269	0.30 ± 0.02	0.24 ± 0.02	p = 0.0473
Distanced moved in Light chamber	F (1, 45) = 0.4760	P = 0.4938	F (1, 45) = 2.862	P = 0.0976	F (1, 45) = 0.6910	P = 0.4102	613.5 ± 40.1	498.8 ± 41.9	p = 0.0141
**Marble Burying**	**Interaction**		**Genotype**		**Gender**		**GGA3KO n = 21**	**GGA3WT n = 28**	**p-value**
No. of marbles buried in 30mins	F (1, 34) = 1.528	P = 0.2248	F (1, 34) = 0.6774	P = 0.4162	F (1, 34) = 0.2426	P = 0.6255	9.1 ± 1	10.4 ± 1	n.s.
**Forced Swim Test**	**Interaction**		**Genotype**		**Gender**		**GGA3KO n = 15**	**GGA3WT n = 23**	**p-value**
Time spent immobile (sec)	F (1, 34) = 0.5082	P = 0.4808	F (1, 34) = 30.72	P < 0.0001	F (1, 34) = 2.667	P = 0.1117	9.7±3.5	43.8±4.4	p<0.0001
**Fear Conditioning**	**Interaction**		**Genotype**		**Gender**		**GGA3KO n = 21**	**GGA3WT n = 28**	**p-value**
24hrs between conditioned stimulus training and test									
Context dependent freezing (%)	F (1, 45) = 4.475	P = 0.0400	F (1, 45) = 0.2923	P = 0.5914	F (1, 45) = 3.036	P = 0.0883	31.3 ± 2.9	30.8 ± 2.0	n.s.
Cued freezing (%)	F (1, 45) = 0.7394	P = 0.3944	F (1, 45) = 0.5993	P = 0.4429	F (1, 45) = 1.657	P = 0.2045	33.1 ± 3.4	35.0 ± 2.1	n.s.

n.s, not significant p-value

#### Neuromotor and Basal Activity Tests

Circadian Activity monitoring: Mice were single housed in transparent standard (20 x 30cm) shoe-box cages with minimal bedding and food and water available ad libitum. Lighting was maintained on the reverse 12:12hrs light:dark cycle and activity was recorded for 24hrs utilizing the Smart Frame^®^ Cage Rack System (Hamilton Kinder, Poway, CA) which consists of 12 photocells (8L x 4W) to track animal movement. Photobeam breaks were monitored and binned utilizing Motor Monitor^®^ software (Hamilton Kinder, Poway, CA) to reveal data including distance travelled (cm) and rest time (sec).

Rotarod: Motor coordination and balance were measured in the mice utilizing an acceleration paradigm on a 5 position Rotarod (ENV 577M, Med Associates, St Albans, VT). The day prior to testing mice were habituated to the Rotarod with two trials (1hr apart) utilizing a slow acceleration paradigm (2-20rpm over 5minutes). Subsequent testing was carried out 24hrs later utilizing the fast acceleration paradigm (4-40rpm over 5minutes). Mice were tested over the course of three trials (1hr apart) and latency to fall (clinging to the cylinder for 2–3 rotations was also classified as a fall) measured in each trial and averaged. Data was reported as RPM at which mice fall utilizing the following equation RPM = [End Speed-Start Speed/300] x (Latency) + Start Speed. Mice with coordination and balance deficits will have shorter latencies to fall during the acceleration paradigm.

#### Exploratory Behavior Tests

**Open Field Testing (OFT):** Individual mice are placed into the open field arena (Plexiglass enclosure, 41L x 41W x 38H cm) surrounded by the 32 photocell Smart Frame Open Field Activity System Frame (Hamilton Kinder), which continuously tracks animal movement over the 5 minutes test period. Photobeam breaks in the center and periphery of the arena are recorded utilizing MotorMonitor software, which provides data including: Time spent in Center and Periphery (sec), distance travelled (cm), center crossings.

#### Cognitive Tests

Morris Water Maze (MWM): This test examines rodents’ spatial reference memory by measuring the ability of the mouse to remember the location of a hidden platform in a water-filled pool through the use of overt spatial cues provided in the testing room in order to escape from the water. The MWM consists of a plastic tub (122cm diameter) filled with opaque (non-toxic white paint stained) water (25°C) to a depth 25cm below the tub rim. The water maze is divided into four quadrants and a highly visible cue is placed on the walls of the testing room corresponding to each of the quadrants. A hidden Plexiglass platform (10cm x 10cm) is submerged 2cm below the water surface in center of a designated quadrant. The mice are placed in the water from one of 4 designated random spatial drop points and given 60sec to find the hidden platform. Mice that are unable to find the platform are guided to it and must remain on the platform for 30sec before being removed to a heated cage to dry off. A total of 8 trials per day over the course of 3 days are utilized during the hidden platform training with an inter-trial interval of 25 minutes. Spatial memory deficits are indicated by longer latencies to find the submerged platform over the course of the training period. 24hrs following the cessation of submerged platform training the platform is removed and mice are subjected to a 60sec probe trial. Mice who have correctly learnt the platform position using the spatial cues provided spend more time in the quadrant that contained the submerged platform and correctly cross the removed platform location more often. During the hidden platform trials and the probe trial, the mouse is tracked using Ethovision video tracking equipment and software (Noldus Bv).

Context and Cued Fear Associated Memory: Delayed fear conditioning is performed to detect any potential deficits in contextual (hippocampus-dependent) or cued (hippocampus and amygdala-dependent) fear memory in mice. Training take place in a clear, 20cm x 20cm by 27.5cm chamber (Stoelting Inc., Wood Dale, IL). The bottom of the chamber consists of a metal rod floor for footshock delivery (San Diego Instruments, San Diego, CA). A camera mounted above the chamber records the test sessions, which are scored for freezing behavior utilizing computer driven software FreezeFrame. A computer is connected to the camera and chamber, and controls the delivery of the unconditioned foot-shock stimulus (0.7mA, 2sec duration, AC current). FreezeFrame computes an arbitrary freezing score to quantify bouts of freezing. Analysis of freezing data was carried out utilizing FreezeView software. For all analysis bout duration was set to 1sec, freezing threshold for the motion index was set to the nadir occurring between 0 and 50. All videos were subsequently watched to confirm that software selected freezing corresponded with video freezing. Training: the mouse was placed in the test chamber and allowed to explore freely for 2min for baseline activity. Following the 2min habituation period, the conditioned stimulus (tone, CS) was played for 30sec at 80 dB. During the last 2sec of the tone, a foot-shock (unconditioned stimulus, US) was delivered which co-terminated with the tone. Mice received a total of two CS-US pairing followed by 2 minutes of no stimulus at the end of training. Contextual fear conditioning was tested 24hrs later in the same chamber. Freezing behavior was scored for 5 minutes, in the absence of CS and US. Cued fear conditioning was conducted 4hrs after the cessation of contextual testing; mice were placed in a novel opaque plastic chamber, which had been thoroughly cleaned with 70% ethanol. Cued testing consisted of a 2 minutes baseline followed by 3 minutes of CS.

#### Repetitive Behavior Test

Marble Burying: The tendency for repetitive behavior is measured in the mice with the marble burying test. Briefly, 22 black marbles (1cm diameter) are placed equidistantly around the edge (1inch from wall) of a standard mouse shoebox cage (20cm x 30cm) on top of 5-inches of hard packed bedding. The mouse is placed in the center of the cage and left to explore the cage for 30 minutes undisturbed. After 30 minutes the mouse is removed and the number of marbles successfully buried are counted. A marble is classified as buried if it was at least 2/3rds covered by bedding. Mice displaying a repetitive behavior phenotype will bury significantly more marbles during the 30 minutes period.

#### Anxiety-like behavior Tests

Elevated Plus Maze (EPM): The fully automated EPM (Hamilton Kinder, Poway, CA) consists of two open arms (38L x 5W cm) and two closed arms (38L x 5W x 15H cm) with a central intersection (5cm x 5cm) forming a cross, which is elevated 75cm above the floor. Movement is detected by 48 equally spaced photocells. EPM exploits two conflicting tendencies in mice; the rodents’ innate drive to explore novel environments and their aversion to heights, hence, mice that are more anxious will spend more time exploring the closed arm. Mice are placed in the center intersection facing an open arm given 5 minutes to explore the maze. Movement through the EPM are monitored utilizing Motor Monitor^®^ software (Hamilton Kinder, Poway, CA) to reveal data including time spent in open and closed arms (sec), number of entries into open and closed arms, distance travelled in open and closed arms (cm), rest time in open and closed arms (sec).

Light/Dark Exploration (L/D): This test exploits rodents’ natural desire to explore closed dark compartments over light open compartments to measure anxiety-related phenotypes. Mice are placed individually into a Plexiglass chamber (44L x 22W x 22H cm) containing two equally sized compartments; one dark completely enclosed except for side opening and the other clear and open. The Plexiglass chamber is surrounded by a PC-interfaced horizontal photobeam frame (SmartFrame^®^ Cage Rack System; Hamilton Kinder), which consists of photocells (8L x 4W cm) that continuously tack the mouse’s movements. Mice are placed in the dark compartment and given 5 minutes to freely explore both chambers. Data revealed through use of Motor Monitor^®^ software (Hamilton Kinder, Poway, CA) include: distance travelled (cm), time spent in dark and light chambers (sec) and number of transitions between chambers.

#### Depressive-like Behavior Test

Porsolt Forced Swim test: Originally designed by Porsolt et al., (1978), it is used to measure depressive-like phenotypes in rodent and exploits the fact that mice forced to swim in a confined environment will display immobility following failed attempts to escape. The greater the level of immobility in mice during this test is considered to indicate increased despair or depressive-like behavior. Mice are placed individually into a glass chamber (height 40cm, diameter 18cm) containing water (30cm depth, 25°C) and allowed to swim for 6 minutes while been video- recorded. Mice are then scored for time spent immobile following the initial 2 minutes habituation period during the 6 minutes trial. Immobility is classified circling floating behavior where only minimal leg movement (i.e. one leg) is present to prevent drowning, with no forward trajectory.

### Electrophysiology experiments

#### Hippocampal slice preparation

Brain slices were prepared from 6 to 8 week old male GGA3KO mice and GGA3WT littermates. Mice were anesthetized with isoflurane, decapitated, and brains were rapidly dissected out and put in an ice-cold cutting solution containing (mM): 126 NaCl, 2.5 KCl, 0.5 CaCl_2_, 2 MgCl_2_, 26 NaHCO_3_, 1.25 NaH_2_PO_4_, 10 glucose, 1.5 sodium pyruvate, and 3 kynurenic acid. Coronal 310μm thick slices were cut with the vibratome VT1000S (Leica Microsystems, St Louis, MO, USA). The slices were then transferred into incubation chamber filled with pre-warmed (31–32°C) oxygenated artificial cerebro-spinal fluid (ACSF) with the following composition (mM): 126 NaCl, 2.5 KCl, 2 CaCl_2_, 2 MgCl_2_, 26 NaHCO_3_, 1.25 NaH_2_PO_4_, 10 glucose, 1.5 sodium pyruvate, 1 glutamine, 3 kynurenic acid and 0.005 GABA bubbled with 95% O_2_−5% CO_2_. Slices were allowed to recover at 32°C for 1h before recording.

#### Electrophysiology Recordings

After recovery, a single slice was transferred to a submerged recording chamber on the stage of an upright microscope (Nikon FN-1) with a 40X water immersion objective equipped with DIC/IR optics. Slices were maintained at 32°C and gravity-superfused with ACSF solution throughout experimentation and perfused at rate of 2 ml/min with oxygenated (O_2_/CO_2_ 95/5%) ACSF. Adequate O_2_ tension and physiological pH (7.3–7.4) were maintained by continually bubbling the media with 95% O_2_/5% CO_2_.

Currents were recorded from the dentate gyrus granule cells (DGGCs) in 310-μm-thick coronal hippocampal slices. Patch pipettes (5–7 MΩ) were pulled from borosilicate glass (World Precision Instruments) and filled with intracellular solution with the following composition (mM): 140 CsCl, 1 MgCl_2_, 0.1 EGTA, 10 HEPES, 2 Mg-ATP, 4 NaCl and 0.3 Na-GTP (pH:7.25). A 5 minutes period for stabilization after obtaining the whole-cell recording conformation (holding potential of -60mV) was allowed before currents were recorded using an Axopatch 200B amplifier (Molecular Devices), low-pass filtered at 2kHz, digitized at 20kHz (Digidata 1440A; Molecular Devices), and stored for off-line analysis.

#### Electrophysiology Analysis

For tonic current measurements, an all-points histogram was plotted for a 10sec period before and during picrotoxin application, once the response reached a plateau level. A Gaussian fit to these points gave the mean current amplitude and the difference between these two values was considered to be the tonic current and normalized to cell capacitance (pA/pF). Series resistance and whole-cell capacitance were continually monitored and compensated throughout the course of the experiment. Recordings were excluded from data analysis if series resistance increased by >20%. Spontaneous IPSCs were analyzed using the mini-analysis software (version 5.6.4; Synaptosoft, Decatur, GA). sIPSCs were recorded for a minimum of 5 minutes. Minimum threshold detection was set to 3 times the value of baseline noise signal. To assess sIPSC kinetics, the recording trace was visually inspected and only events with a stable baseline, sharp rising phase, and single peak were used to negate artifacts due to event summation. Only recordings with a minimum of 100 events fitting these criteria were analyzed. Difference in amplitude distributions of spontaneous currents obtained from a single neuron was examined by constructing all-point cumulative probability distributions.

### Quantification of inhibitory synapses

#### Immunohistochemistry

Three month-old male GGA3WT and GGA3KO mice were perfused intracardially with PBS (0.1M, pH:7.4) followed by 4% PFA in PBS. Brains were removed and post-fixed with 4% PFA in PBS for 2hrs, at 4°C. The next day, the brains were washed 3 times in PBS and then cryoprotected with 30% sucrose in PBS. Coronal sections of 50μm where sliced using a Leica SM2010R sliding microtome. Antigen retrieval was performed by incubating free floating sections in 10mM sodium citrate buffer (pH 6.0) preheated at 60°C for 35min, and later brought to room temperature and rinsed in PBS. Non-specific binding sites were blocked for 2hrs at room temperature (5% BSA, 0.3% Triton-X-100 in PBS). Sections were then incubated with the following primary antibodies (diluted in 2% BSA, 0.3% Triton-X-100 in PBS): rabbit polyclonal anti-vGAT (1:2000, Cat.No. 131 003, Synaptic System, Germany) and mouse monoclonal anti-gephyrin (1:400, Cat.No 147 111, Synaptic System, Germany) for 70 hours at 4°C). The specificity of anti-vGAT and anti-gephyrin antibodies has been previously demonstrated [20, 21]. Next, the sections were incubated with the appropriate secondary antibodies (goat anti-rabbit IgG Alexa Fluor^®^ 488 and goat anti-mouse IgG Alexa Fluor^®^ 594, Invitrogen, USA) for 2hrs at RT (1:500 in 2% BSA, 0.3% triton-X-100 in PBS). Sections where finally washed in PBS and mounted on superfrost slides using Fluoromount-G^®^ mounting media (Southern Biotech, USA).

#### Synapse quantification

The synapse quantification was performed according to a well-established protocol [22]. Synaptic quantification of inhibitory synapses was performed in the dentate gyrus using two coronal brain sections per animal (4 males GGA3WT and 6 males GGA3KO) immunostained with pre- and post-synaptic markers (vGAT and gephyrin, respectively) and 5-μm confocal scans were performed (optical section width, 0.34μm; 15 optical sections each) using a 63X/NA 1.4 immersion oil objective on a LEICA SPE confocal microscope equipped with a spectral scanner system (range of detection from 400 to 850nm). The acquisition settings were set up on GGA3WT brain sections, and maintained constant for GGA3KO mice. Three consecutive optical sections were merged, to obtain a z-stack projection of ~ 1μm, and analyzed using the ImageJ (v1.29, NIH) Puncta Analyzer Plug-in Application, provided by Dr. C. Eroglu, as previously described [22]. Synapses were defined as vGAT and gephyrin positive co-localizing puncta. The number of pre- and postsynaptic co-localizing puncta were counted and expressed as total number of co-localizing puncta per 5μm depth z-stack image.

### Statistical Analysis

GraphPad Prism 6 was used for statistical analysis. For all behavioral analysis with the exception of MWM a two-way ANOVA was used to compare genotype, gender and their interaction. A two-tailed Student’s *t*-test was used for post-hoc analysis to compare only the effect of genotype as this comparison was determined *a priori* to be the one of interest. Data sets consisting of two-groups, which passed normality and Bartlett’s test, were analyzed using an unpaired *t*-test, where standard deviations between groups were significantly different a Welch correction was employed. Two-group data sets that demonstrated non-normal distribution were analyzed by Mann-Whitney test. For the analysis of the MWM test, SPSS software (IBM inc) was used to perform a 3-way analysis of variance with the following independent variables: Genotype, Gender, and Day of experiment. In the analysis the Day variable was treated as repeated measures, while the other two, Gender and Genotype, where not. Interaction factors were included for interactions between all independent variables. Two-way ANOVA with repeated measures was used to compare genotype and days of training.

## Results

### GGA3KO mice show normal basal activity and motor coordination

Monitoring of circadian locomotor activity over a period of 24 hr to reveal basal activity levels demonstrated no overt differences in basal activity between GGA3 null (GGA3KO) and their WT littermates as no statistically significant differences were observed in total distance moved or total rest time between GGA3KO and GGA3WT mice over a 24 hr period (Table 1). Furthermore, no differences were observed in gross motor performance between GGA3KO and GGA3WT littermates as measured by rotarod monitoring assay (Table 1).

### GGA3KO mice exhibit enhanced exploratory behavior and novelty-induced hyperactivity

**Fig 1:**
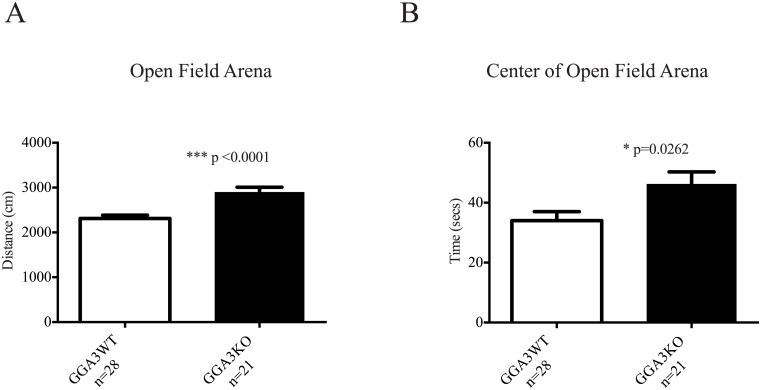
GGA3KO mice exhibit enhanced exploratory behavior and novelty-induced hyperactivity. GGA3KO and GGA3WT mice were subjected to the Open Field Test (OFT). GGA3 deletion induced enhanced exploratory behavior and hyperactivity as demonstrated by increased locomotor activity (A) and increased time spent exploring the center of the arena (B). The absence of differences in basal activity and locomotion in the circadian activity monitoring test indicates that the hyperactivity phenotype witnessed in GGA3KO mice is novelty-induced. The graphs represent mean ± SEM. n = 21 GGA3KO mice; n = 28 GGA3WT mice of mixed gender. The *t*-test was used for statistical analysis and * indicate a p-value <0.05.

Open Field Testing (OFT) of GGA3KO and GGA3WT mice revealed that GGA3 deletion was correlated with increased locomotor activity (Table 1, Fig 1A). This increased locomotion in GGA3KO versus their wild-type counterparts in the open field arena was in direct contrast to the comparative locomotor and basal activity witnessed in GGA3KO and WT mice in circadian activity testing. Taken together, the selective hyperactivity phenotype witnessed in GGA3KO mice only in the open field task indicates that it is novelty-induced. In addition to the increased locomotor activity, GGA3KO mice spend increased time in the center of the field (Fig 1B) and trend towards increased exploration of the center of the open field arena (% of total path length in center GGA3KO 0.219 ± 0.013, GGA3WT 0.184 ± 0.011, p = 0.0518) which taken together with increased locomotion indicates increased exploratory behavior in GGA3KO mice compared to their WT counterparts.

### GGA3KO mice do not exhibit cognitive deficits or altered repetitive behaviors

**Fig 2:**
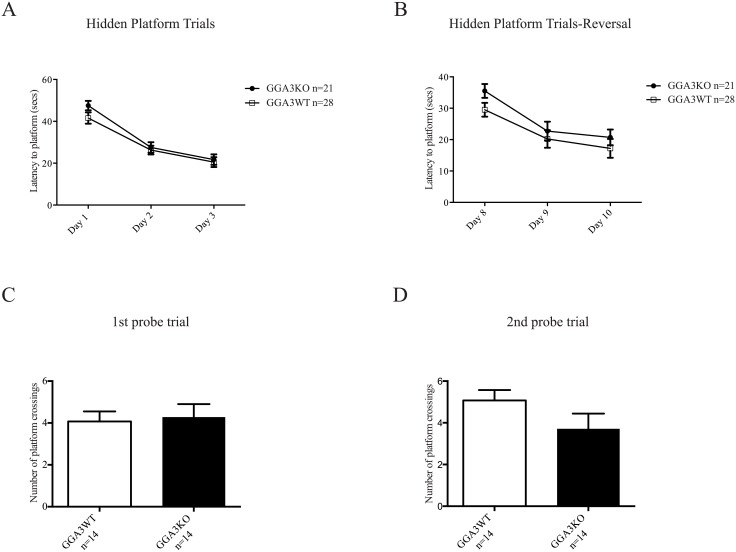
GGA3KO mice do not exhibit deficits in spatial reference memory. GGA3KO and GGA3WT were subjected to Morris Water Maze test. Both groups of mice demonstrate a significant decrease in latency to find the hidden platform (8 trials/day; 25min ITI) in initial testing (Days 1–3) phase (A) and during reversal phase (Days 8–10) (B) (Days 1–3 ***p value <0.001, Days 8–10 ***p value <0.001) however no significant difference in latency is observed between GGA3KO and WT mice on any day of testing. No significant differences were observed in the number of platform crossings during the 1^st^ or 2^nd^ probe trial (C-D). The graphs represent mean ± SEM. n = 14 GGA3KO mice and n = 14 GGA3WT mice of mixed gender. Two-way ANOVA repeated measures and *t*-test were used for latency to platform and probe trials, respectively.

GGA3KO mice did not exhibit deficits in spatial reference memory as tested by Morris water maze including a reversal analysis (Fig 2A–2D, Tables 2 and 3) and in contextual (hippocampus-dependent) or cued (hippocampus and amygdala-dependent) fear associated memory (Table 1). Furthermore, GGA3KO mice did not demonstrate any differences in repetitive behaviors as measured by the marble burying test when compared to their WT littermates (Table 1).

**Table 2:** 

			Three way ANOVA						Two way ANOVA repeated measures			
Morris Water Maze	Interaction		Genotype		Gender		Interaction		Genotype		Day	
Hidden platform trials (8 trials per day)												
Latency to Platform Day 1–3 (sec)	F (1,24) = 0.176	P = 0.679	F (1,24) = 1.323	P = 0.261	F (1,24) = 6.388	P = 0.018	F (2, 52) = 0.9605	P = 0.3894	F (1, 26) = 1.125	P = 0.2985	F (2, 52) = 77.48	P < 0.0001
Swim speed Day 1–3 (cm/s)	F (1,24) = 0.684	P = 0.416	F (1,24) = 2.622	P = 0.118	F (1,24) = 0.803	P = 0.379	F (2, 52) = 1.323	P = 0.2750	F (1, 26) = 2.674	P = 0.1140	F (2, 52) = 61.10	P < 0.0001
Reversal hidden platform trial (8 trials per day)												
Latency to Platform Day 8–10 (sec)	F (1,24) = 0.134	P = 0.717	F (1,24) = 1.776	P = 0.195	F (1,24) = 0.683	P = 0.417	F (2, 52) = 0.4432	P = 0.6444	F (1, 26) = 1.860	P = 0.1843	F (2, 52) = 28.15	P < 0.0001
Swim speed Day 8–10 (cm/s)	F (1,24) = 0.488	P = 0.492	F (1,24) = 3.739	P = 0.065	F (1,24) = 0.525	P = 0.476	F (2, 52) = 1.410	P = 0.2533	F (1, 26) = 2.338	P = 0.1383	F (2, 52) = 34.83	P < 0.0001

**Table 3:** 

	Two way ANOVA							*t*-test	
1st Probe trial (24hrs following hidden platform training)	**Interaction**		**Genotype**		**Gender**		**GGA3KO n = 14**	**GGA3WT n = 14**	**p-value**
Time spent in correct quadrant (sec)	F (1, 24) = 0.289	P = 0.595	F (1, 24) = 0.450	P = 0.508	F (1, 24) = 0.942	P = 0.341	22.7 ± 2.1	24.5 ± 1.8	n.s.
No. of platform crossings	F (1, 24) = 0.007	P = 0.931	F (1, 24) = 0.068	P = 0.795	F (1, 24) = 0.375	P = 0.546	4.3 ± 0.6	4.1 ± 0.5	n.s.
2nd Probe trial (24hrs following reversal training)									
Time spent in correct quadrant (secs)	F (1, 24) = 0.107	P = 0.745	F (1, 24) = 0.021	P = 0.885	F (1, 24) = 4.647	P = 0.041	23.1 ± 1.9	22.7 ± 2.3	n.s.
No. of platform crossings	F (1, 24) = 0.441	P = 0.512	F (1, 24) = 3.252	P = 0.083	F (1, 24) = 12.33	P = 0.001	3.7 ± 0.8	5.1 ± 0.5	n.s.

n.s, not significant p-value

### GGA3 deletion induces a reduction in anxiety-like behaviors

**Fig 3:**
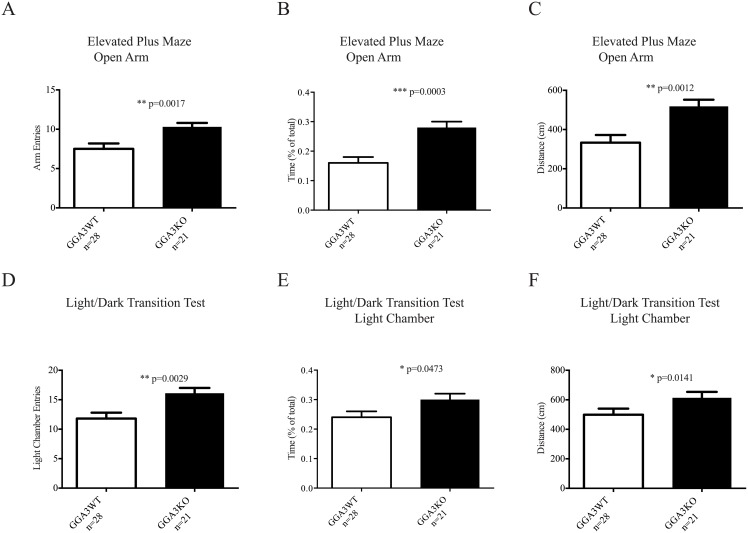
GGA3 deletion induces a reduction in anxiety-like behaviors. GGA3 null mice display decreased anxiety-like behaviors in two different testing paradigms (A-C: Elevated Plus Maze; D-F: Light-Dark Transition). GGA3KO mice entered more frequently (A), spent significantly more time (B) and explored more (C) the open-arm of the elevated-plus maze in a 5 minute test than their WT littermates. GGA3KO mice entered more frequently (D), spent significantly more time (E) and explored more (F) the light chamber of the light/dark transition apparatus in a 5 minutes test than their WT littermates. The graphs represent mean ± SEM. n = 21 GGA3KO mice and n = 28 GGA3WT mice of mixed gender were used in the both tests. The *t*-test was used for statistical analysis and * indicate a p-value <0.05.

Anxiety–like behaviors were assessed with two separate behavioral paradigms: elevated plus maze (EPM) and the light/dark transition tests (L/D). In the elevated plus maze GGA3KO mice entered the open arm more frequently (Fig 3A), spent significantly more time in the open arm (Fig 3B) and explored the open arm of the maze more (Fig 3C) than their GGA3WT littermates. Furthermore, in the light/dark transition test GGA3KO mice entered the light chamber more often (Fig 3D), spent significantly more time in the light chamber (Fig 3E) and demonstrated a greater exploration of the light chamber (Fig 3F). Thus, GGA3KO mice demonstrated reduced anxiety-like behaviors in both tests (Table 1). Moreover, this phenotype was independent of increased locomotor activity or hyperactivity as the path length was similar in GGA3WT and GGA3KO mice in both elevated plus maze and light dark/transition (EPM: 1752.9 ± 36.4 vs 1827.1 ± 49.6 and L/D:1755±80 and 1986±95, respectively).

### GGA3 deletion reduces depressive-like behaviors

**Fig 4:**
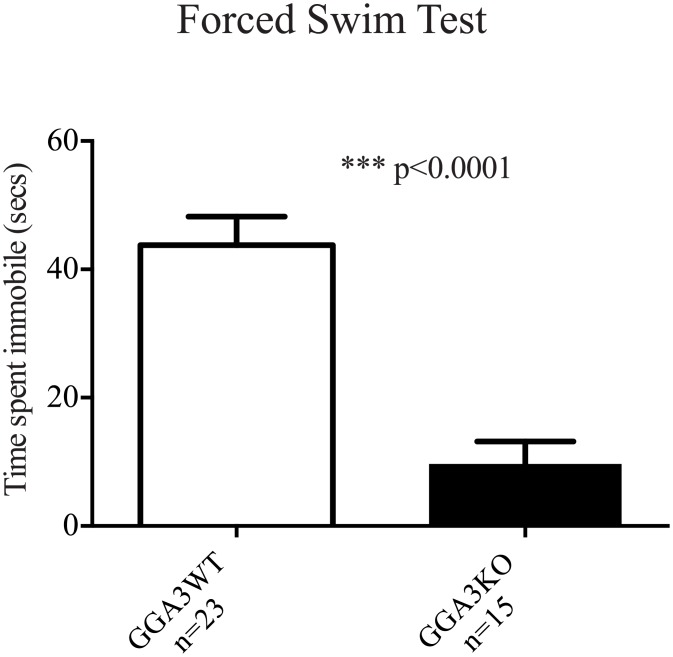
GGA3 deletion induces an anti-depressive-like phenotype. GGA3KO and GGA3KO were subjected to the Porsolt Forced Swim Test and demonstrated significantly reduced immobility in compared to their WT littermates during a six minutes test. The graph represents mean ± SEM. n = 15 GGA3KO mice and n = 23 GGA3WT mice of mixed gender were used. The *t*-test was used for statistical analysis and * indicate a p-value <0.05.

In light of the reduction in anxiety-like behaviors in GGA3KO mice, GGA3KO and GGA3WT were subjected to the Porsolt Forced Swim Test. GGA3KO mice demonstrated significantly reduced immobility in compared to their WT littermates (Fig 4). The Porsolt Forced Swim Test was originally developed to determine the effectiveness of acute anti-depressant administration on learned helplessness/depressive-like behavior in rodents. The significant reduction in immobility in GGA3KO mice provides further evidence of a potential role for GGA3 in emotional and motivational behaviors. However, as was noted in open-field testing, the predisposition of GGA3KO mice to novelty induced hyperactivity and increased locomotion potentially confounds this behavioral phenotype and thus it was not explored further.

### GGA3KO mice display an increase in phasic inhibition and decreased tonic current

**Fig 5:**
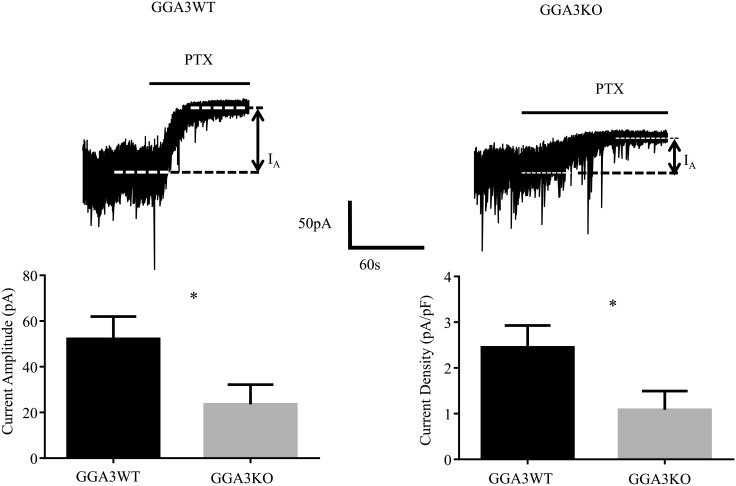
GGA3KO mice display deficits in GABAergic tonic current. Recordings were made from DGGCs in hippocampal slices from 6–8 week-old GGA3WT and GGA3KO mice in the presence of 1μM GABA. Tonic current was determined by measuring the difference in holding current amplitude before and after applying 100 μM picrotoxin (PTX). GGA3KO mice exhibited a significant reduction in tonic current amplitude (A). The graph represents mean ± SEM of current amplitude and density (B). * = significantly different to control (p<0.05; *t*-test, n = 7–8 cells, from 3 animal for genotype).

**Fig 6:**
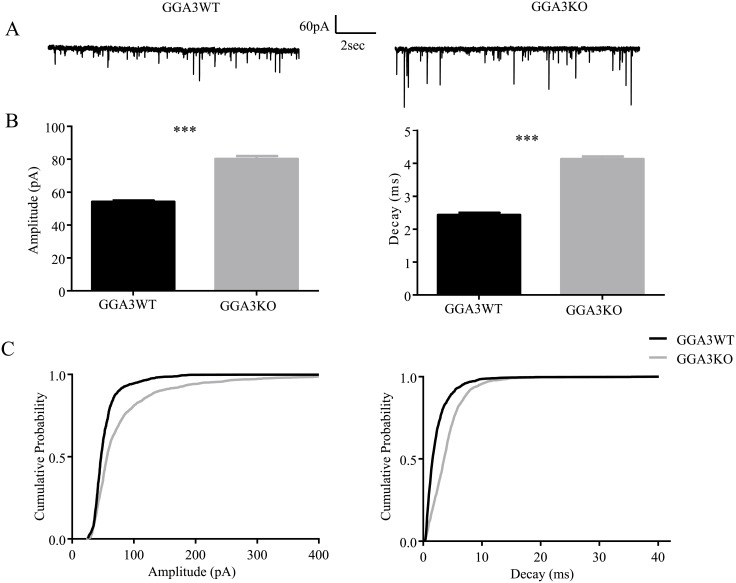
GGA3KO mice display increased GABAergic phasic current. Recordings were made from DGGCs in hippocampal slices from 6–8 week-old GGA3WT and GGA3KO mice. GGA3KO mice displayed larger sIPSC amplitudes and longer decay times compared to age-matched controls (A-B) as seen in the cumulative probabilities for sIPSC amplitude and decay (C). The graph represents mean ± SEM of sIPSC amplitude and decay from GGA3WT and GGA3KO mice (*** p<0.0001 Mann-Whitney test from 6 cells from 3 animals for genotype).

Given that GABAergic inhibition is implicated in anxiety and depressive behaviors [19], we next compared the properties of synaptic and extrasynaptic inhibitory currents in DGGCs from GGA3KO and GGA3WT mice. We decided to perform recording from the DGGCs given that both GGA3 and BACE1 are highly expressed in this region [17, 23]. Patch-clamp recordings were used to analyze GABAergic inhibition from the DGGCs from GGA3KO and GGA3WT and revealed that phasic and tonic properties were altered in GGA3KO mice. In DGGCs from GGA3KO mice, tonic current was significantly decreased (52.19 ± 9.768 vs. 23.55 ± 8.62 pA, for GGA3WT and GGA3KO littermates respectively, p< 0.05, n = 7–8 cells, 3 mice of each genotype, Fig 5). In contrast, the amplitude of sIPSCs was significantly increased in GGA3KO compared to GGA3WT (54.21± 0.71 vs. 80.24 ± 1.69 pA, for GGA3WT and GGA3KO, respectively, p <0.005, n = 7–8 cells, 3 mice of each genotype, *t*-test). Moreover, the decay time of sIPSCs (2.43 ± 0.06 vs. 4.13 ± 0.07 s, p > 0.0001, n = 6 cells, 3 mice of each genotype for GGA3WT and GGA3KO mice, respectively) was significantly slowed in GGA3KO mice (Fig 6A–6C) but, the inter-event interval of sIPSCs was comparable between GGA3WT and GGA3KO genotype (299.1 ± 8.743 vs. 318.6 ± 13.68 ms, p>0.05, n = 6 cells, 3 mice of each genotype for GGA3WT and GGA3KO mice, respectively). Collectively, these results indicate that GGA3KO mice display reduced tonic but enhanced phasic inhibition.

### The number of inhibitory synapses is increased in the dentate gyrus of GGA3KO mice

**Fig 7:**
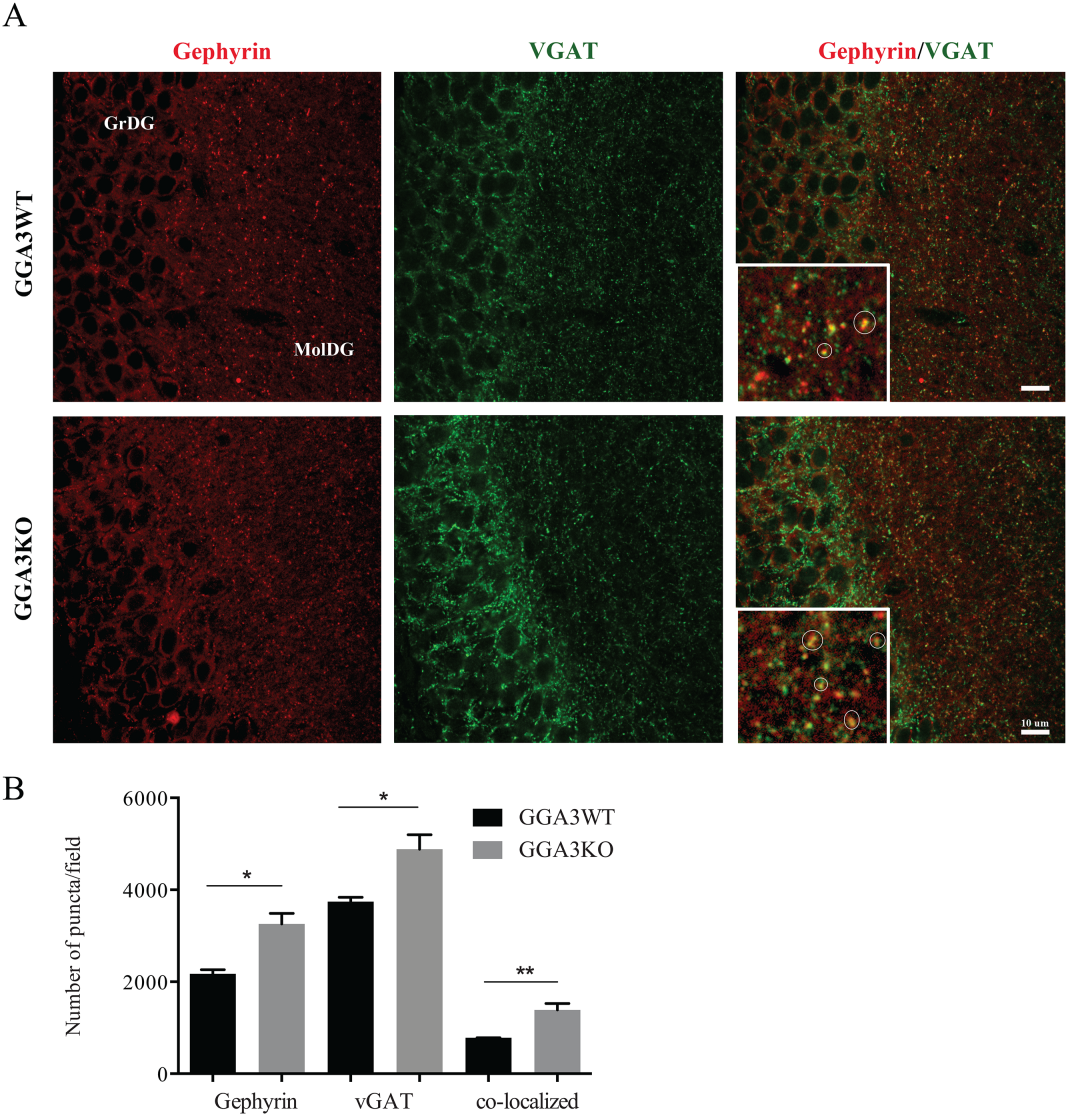
The number of inhibitory synapses is increased in the dentate gyrus of GGA3KO mice. (A) 50 μm coronal brain slices were stained with pre- and post-synaptic marker antibodies (vGAT and gephyrin, respectively) and fluorescent conjugated secondary antibodies, followed by confocal microscopy. The numbers of vGAT (green, left panels), gephyrin (red, middle panels), and co-localizing puncta (vGAT/gephyrin, right panels) were quantified in the dentate gyrus of GGA3WT (upper panels) and GGA3KO mice (lower panels). A magnified image of the molecular granular layer of the dentate gyrus (MolDG) it is shown in the inset of the right panels, where the co-localizing puncta are highlighted within a circle. GGA3KO mice showed an increase in the presynaptic, postsynaptic, and co-localizing puncta. GrDG: Granular cell layer, MolDG: Molecular layer. Scale bar 10μm. (B) The graph represents mean ± SEM of the total number of puncta measured in 4 GGA3WT and 6 GGA3KO mice using Puncta Analyzer Plug-in. Two-four optical fields (116.4 x 114.4 x 5 μm) from each of two brain sections were analyzed for each mouse. The number of gephyrin, vGAT and co-localizing puncta were increased in GGA3KO mice compared to the wild-type littermates. Mann-Whitney test was used for statistical analysis, * p<0.05, **p = 0.0095.

Since increased GABAergic inhibition could results from an increased number of GABAergic synapses, we next determined the number of the inhibitory synapses (defined as gephyrin/vGAT co-localizing puncta) in the dentate gyrus. Hippocampal sections were immunostained using pre- and post-synaptic marker antibodies (vGAT and gephyrin, respectively) and images acquired by confocal microscopy (Fig 7A). The number of gephyrin, vGAT, and co-localizing puncta (vGAT/gephyrin) were quantified in the dentate gyrus of GGA3WT (Fig 7A) and GGA3KO mice (Fig 7A) as previously described [22, 24]. A magnified image of the molecular granular layer of the dentate gyrus (MolDG) it is shown in the inset on the right panels, where the co-localized puncta are highlighted within a circle. The number of gephyrin, vGAT and co-localizing puncta/synapses were increased in GGA3KO mice (Fig 7B) compared to GGA3WT littermates. These findings are in further support of the electrophysiology data and strongly suggest that GGA3 plays a pivotal role in GABAergic transmission.

## Discussion

Polarized delivery of membrane proteins is regulated by the interaction of signals present in their carboxyl-terminal fragment (CTF) with specific trafficking molecules [25]. Sorting signals include the di-leucine-based motifs, [DE]XXXL[LI] or DXXLL, the tyrosine-based motifs, NPXY or YXX∅, and ubiquitin [26]. The [DE]XXXL[LI] motif is recognized by the adaptor protein (AP) complexes AP-1, 2, 3 and 4, while GGA1, 2, and 3 bind to DXXLL via the VHS domain. While increasing evidence is accumulating for a role of the AP complexes in neuronal polarized sorting [27–29], the function of GGAs in neurons remains to be clarified. We report here the first behavioral characterization of GGA3 null mice. We found that genetic deletion of GGA3 results in novelty-induced hyperactivity and decreased anxiety-like behaviors. In order to identify the underlying mechanism(s) of these behavioral phenotypes, we studied GABAergic transmission given its key role in anxiety- and depressive-like behaviors [19]. Patch-clamp recordings in DGGCs revealed that phasic GABA inhibition was increased, while tonic GABA inhibition was decreased. Moreover, we found that the number of inhibitory synapses was increased in the GGA3 null mice. Thus, it is apparent that GGA3 deficient mice have elevations in phasic inhibition, increased number of inhibitory synapses events and reduced anxiety-like behaviors. Significantly these phenotypes are similar to those seen in mice in which tyrosines 365/7 (Y365/7F) in the GABA_A_R γ2 subunit have been mutated to alanines. This mutation slows receptor endocytosis by decreasing clathrin-AP2 binding leading to an increase in size and number of inhibitory synapses, which correlates with a reduced depressive-like phenotype [30–32]. Moreover mice with reduced levels of the γ2 subunit have enhanced anxiety- and depressive-like behaviors [33, 34].

The mechanisms by which GGA3 modulates the formation and/or activity of inhibitory synapses remains speculative, however it is important to note that GABA_A_Rs undergo ubiquitin-dependent lysosomal targeting which is dependent upon 4 sequential lysine residues in the intracellular domain of the γ2 subunit. Critically this mechanism has been established to regulate the GABA_A_R number at inhibitory synapses together with the efficacy of phasic inhibition [35]. Thus GGA3 may participate in the regulation of GABA_A_R endocytic sorting. Consistent with this notion the γ2L subunit variant contains a di-leucine motif downstream of the ubiquitinated lysine residues that may facilitate the interaction of GABA_A_Rs with GGA3. Accordingly our results reveal that ablating GGA3 expression increases the number of inhibitory synapses and the efficacy of phasic inhibition. We also noted a decrease in the efficacy of tonic inhibition in the GGA3 KO mice. It is emerging that there appears to be a reciprocal relationship between the efficacy of phasic and tonic inhibition. In particular male mice expressing the GABA_A_R γ2 Y365/7F mutant have increased phasic but decreased tonic inhibition. Likewise mice in which serine residues 408/9 in the β3 subunit have been mutated to alanines show similar changes in the equilibrium between phasic and tonic inhibition [31, 36, 37]. However the physiological significance of these changes remains to be addressed.

In addition to a direct role of GGA3 in GABA_A_R degradation, it is also possible that at least some of the phenotypes displayed by the GGA3 null mice are the consequence of the BACE1 elevation observed in the brain of GGA3 null mice [17]. Accordingly, BACE1 transgenic mice exhibit a bolder, less anxious phenotype compared to non-transgenic mice [38, 39]. However, the mechanisms underlying the behavioral phenotypes of the BACE1 transgenic mice remain to be clarified. In contrast, the variety of phenotypes described in the BACE1 null mice is linked to BACE1 multiple substrates [40]. A well-studied BACE1 substrate is Neuregulin-1 (NRG1). NRG are a family of growth and differentiation factors with a wide range of functions in the nervous system. NRG proteins are ligands for the ErbB family of receptor tyrosine kinases [41, 42]. Lack of BACE1 processing of NRG1 has been proposed to be responsible for the hyperactivity and schizophrenia endophenotypes, spine density reduction, myelination deficits in central and peripheral nervous system and deficits in formation and maturation of muscle spindles observed in BACE1 null mice [42]. Interestingly, NRG1 has also been shown to promote GABAergic differentiation and synaptogenesis by interacting with its receptor Erbb4 [43]. Moreover, administration of exogenous NRG1 reduces anxiety-like behaviors and increases GABAergic transmission in mice [44]. Thus, it possible that increased BACE1-mediated processing of NRG1 could lead to increased GABAergic transmission and reduced anxiety.

Additional BACE1 substrates have been identified by quantitative proteomics analysis [45–47] raising the possibility that the increased BACE1-mediated cleavage of other molecules can contribute to the phenotypes observed in the GGA3 null mice. For example, neuroligin-2, a member of a family of postsynaptic cell-adhesion molecules [48], has been identified as BACE1 substrates [46]. Most notably, the overexpression of neuroligin-2 has been shown to drive postsynaptic differentiation of inhibitory synapses [49].

Several synaptic alterations have been described in BACE1 null mice: an increase in the Pair Pulse facilitation (PPF) in the CA1 and CA3 regions of the hippocampus [50–52]; no change in LTP nor in LTD, in CA1 region of the hippocampus, but decreased mossy fiber LTP (mfLTP) and increased in LTD in the CA3 region, approximately 10% more compare with wild-type mice [51–53]. However, it remains unknown the extent to which deletion or overexpression of BACE1 produces changes in GABAergic transmission.

BACE1 is a stress-related protease that is upregulated in AD brains and following acute brain injuries [54]. Our previous studies have established that depletion of GGA3 and BACE1 elevation occurs in AD and acute brain injuries. We originally showed that GGA3 levels are significantly decreased and inversely correlated with BACE1 levels in the post-mortem temporal cortex of patients with AD [15]. Our findings have been confirmed by two independent studies conducted in AD brain samples of Australian and European origin [55] (US Patent Application 20120276076, Annaert, Wim; et al. November 1, 2012). Moreover, we have determined that GGA3 is depleted while BACE1 levels increase following experimental stroke and traumatic brain injury (TBI) [15, 17]. Thus, the phenotypes observed in the GGA3 null mice could help to explain at least some of the symptoms observed in AD patients and TBI sufferers. Patients with Alzheimer’s disease are subject to anxiety, but sometimes also to the opposite tendency of disinhibition [56–62]. Similarly, neuropsychiatric sequelae of TBI include anxiety disorders but also dishinibition and risk-taking behaviors [63–65]. Reduced anxiety and increased risk-taking behavior have been observed in mice post-TBI [66, 67] and in 5XFAD and other mouse models of AD pathology [68–70]. Furthermore, alterations of GABAergic transmission have been detected in mouse models of AD [71] and could be the underlying mechanism(s) of seizures observed in mouse models and AD patients [72]. Similarly, alterations of GABAergic transmission have been implicated in post-traumatic epilepsy [73].

Further studies will be necessary to dissect the role of BACE1 elevation from GGA3 depletion under normal and pathological conditions.
